# Zinc inhibits osteoclast differentiation by suppression of Ca^2+^-Calcineurin-NFATc1 signaling pathway

**DOI:** 10.1186/1478-811X-11-74

**Published:** 2013-10-02

**Authors:** Kwang Hwan Park, Boryung Park, Dong Suk Yoon, Seung-Hyun Kwon, Dong Min Shin, Jin Woo Lee, Hyun Gyu Lee, Jae-Hyuck Shim, Jeon Han Park, Jae Myun Lee

**Affiliations:** 1 Department of Microbiology, Yonsei University College of Medicine, Seoul, Republic of Korea; 2 Department of Orthopaedic Surgery, Yonsei University College of Medicine, Seoul, Republic of Korea; 3 Department of Oral Biology, Yonsei University College of Dentistry, Seoul, Republic of Korea; 4 Brain Korea 21 Plus Project for Medical Sciences, Yonsei University College of Medicine, Seoul, Republic of Korea; 5 Department of Pathology and Laboratory medicine, Weill Cornell Medical College, New York, NY, USA

**Keywords:** Zinc, Bone loss, Osteoclast, NFATc1, Calcineurin, Ca^2+^ oscillation

## Abstract

**Background:**

Zinc, an essential trace element, inhibits osteoclast differentiation in vitro and in vivo. The molecular mechanism for the inhibitory effect of zinc, however, is poorly understood. The purpose of this study was to investigate the effect of zinc and determine its molecular mechanism on receptor activator of NF-κB ligand (RANKL)-induced osteoclastogenesis in mouse bone marrow-derived monocyte cells (BMMs) and RAW264.7 cells.

**Results:**

In BMMs, zinc treatment during osteoclast differentiation decreased RANKL-induced osteoclast formation in a dose-dependent manner. We show that zinc suppressed the mRNA levels of nuclear factor of activated T-cells, cytoplasmic 1 (Nfatc1). Zinc also accumulated phospho-Nfatc1 (p-Nfatc1) in the cytosol in a dose-dependent manner and inhibited the translocation of Nfatc1 to the nucleus in RAW264.7 cells. Zinc suppressed the activities of Nfatc1 in the nucleus without changing the activities of NF-κB in RAW264.7 cells. In contrast, calcineurin activity decreased in response to zinc but its protein level was unchanged. RANKL-induced Ca^2+^ oscillations were inhibited by zinc treatment, but phospho-phospholipase Cγ1 (p-PLCγ1), the upstream signaling molecule of Ca^2+^ oscillations, was unaffected. Moreover, a constitutively active form of Nfatc1 obviously rescued suppression of osteoclastogenesis by zinc.

**Conclusions:**

Taken together, these results demonstrate for the first time that the inhibitory effect of zinc during osteoclastogesis is caused by suppressing the Ca^2+^-Calcineurin-NFATc1 signaling pathway. Thus, zinc may be a useful therapeutic candidate for the prevention of bone loss caused by NFATc1 activation in osteoclasts.

## Background

The balance between osteoclastogenesis and osteoblastogenesis is important for the maintenance of bone homeostasis [1-6]. In particular, bone resorption by osteoclasts is involved in various skeletal diseases, such as osteoporosis and arthritis. There have been many studies about the various genes that are regulated during osteoclastogenesis. Representative up-regulated genes are *Nfatc1*, *Fos*, *Oscar*, and *Ctsk* and down-regulated genes include *Id*, *Mafb*, *Irf8*, and *Bcl6*[7-13].

To identify novel genes involved in osteoclastogenesis, we used two sets of microarray data from Gene Expression Omnibus (GEO) DataSets, which were comparative microarrays in mouse bone marrow-derived monocyte cells (BMMs) stimulated with or without receptor activator of NF-κB ligand (RANKL) [12,13]. We performed statistical data analyses using the R program. From these analyses, we found intersections between the two sets of data. Among the intersections, *Mt3*, which is known to regulate the intracellular level of zinc, and other zinc-related genes were up-regulated (log_2_ ratio > 4.0) during osteoclast differentiation (Additional file 1: Table S1). In literatures, one report showed that dietary zinc and Metallothionein (MT) interact in postnatal bone growth [14]. Also, Lee et al. reported that zinc regulates T cell receptor signaling [15]. We thus suggest that zinc may play an important physiologic role in osteoclastogenesis signaling pathways.

Zinc is an important trace element for biological signaling pathways, but also acts as a second messenger in cells [16]. Zinc supplementation has been reported to inhibit bone loss in an adjuvant-induced rheumatoid arthritis rat model, promoting bone formation and suppressing bone resorption [17]. Dietary zinc is also reported to reduce levels of tartrate-resistant acid phosphatase (TRAP), which is a specific marker of osteoclasts in the tibia and calvaria in vivo [18]. In humans, zinc intake negatively correlates with bone loss in postmenopausal women [19] and positively correlates with bone mass in premenopausal women [20,21]. Despite persuasive studies that zinc is involved in bone loss by suppressing osteoclast differentiation, the molecular mechanism for the inhibitory effect of zinc on osteoclast differentiation remains poorly understood.

In this study, to investigate the molecular mechanism of the inhibitory effect of zinc on RANKL-induced osteoclast differentiation, we focused on Nfatc1, a master transcription factor of osteoclastogenesis [9,13,22-24]. Previously, Mackenzie et al. reported that extracellular zinc can regulate NFAT activity in neuronal cells. So, we confirmed previously reported findings that zinc suppresses osteoclast differentiation in vitro. We discerned that its inhibitory mechanism was involved in blocking the Ca^2+^-Calcineurin-NFATc1 signaling pathway.

## Results

### Zinc inhibits osteoclast formation and fusion from BMMs

**Figure 1:**
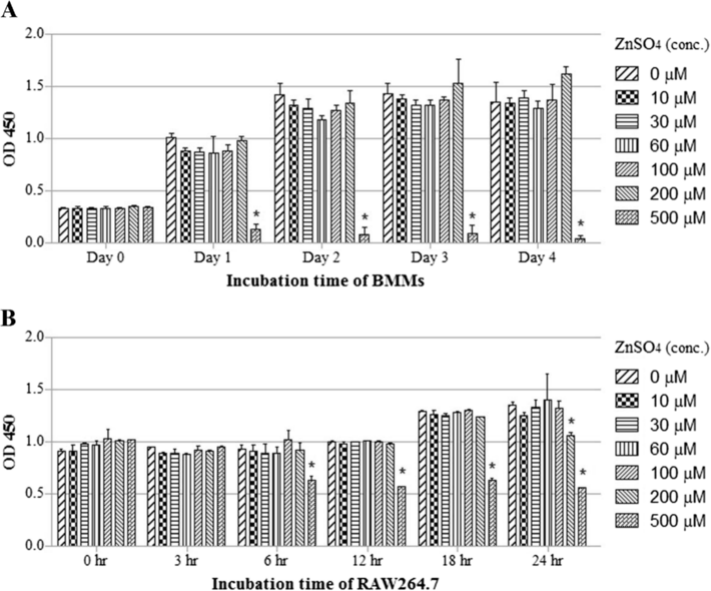
**Measurement of cell viability. (A)** BMMs were cultured with M-CSF (30 ng/ml) and various concentrations of ZnSO_4_ for 4 days. **(B)** RAW264.7 cells were cultured with various concentration of ZnSO_4_ for 24 s. Cell viability was measured using EZcytox cell viability assay kits. Data are presented as the mean ± S.D. of three independent experiments. *P < 0.05 compared to control.

**Figure 2:**
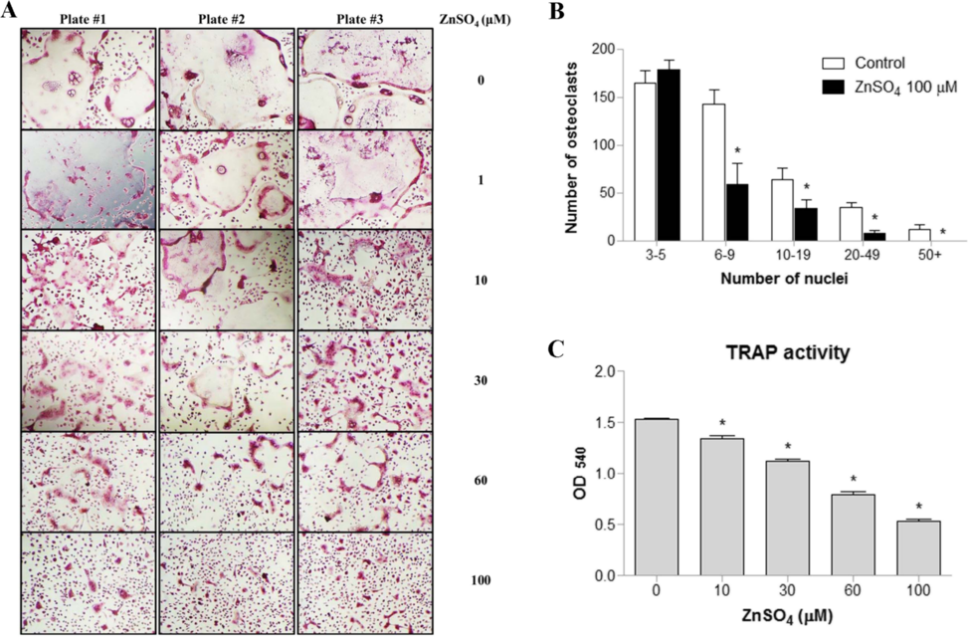
**Zinc inhibits RANKL-induced osteoclast formation and fusion from BMMs. (A)** BMMs were cultured for 4 days with M-CSF (30 ng/ml), RANKL (120 ng/ml), and various concentrations of ZnSO_4_. The cells were stained for TRAP. **(B)** TRAP-positive multinucleated cells (nuclei ≥ 3) were counted using manual counting and a nuclei-counter plug-in for the Image J program. **(C)** TRAP activity was measured at 540 nm. Data are presented as the mean ± S.D. of three independent experiments; *P < 0.05 compared to control.

To determine whether zinc is cytotoxic to BMMs and RAW264.7 cells, we first examined the cells’ viability using an EZcytox cell viability assay kit, which estimates the number of surviving cells using WST-1 (4-[3-(4-iodophenyl)-2-(4-nitrophenyl)-2H-5-tetrazolio]-1,3-benzene disulfonate). BMMs and RAW264.7 cells, which were each treated with up to 200 and 100 μM ZnSO_4_, were viable for 4 days and 24 hours, respectively (Figure 1). We thus designated 100 μM ZnSO_4_ as the maximal concentration that was nontoxic to both BMMs and RAW264.7 cells. We investigated the effects of zinc on osteoclast formation of BMMs in vitro by treating BMMs with M-CSF and RANKL in the presence or absence of zinc. Zinc treatment inhibited osteoclast formation in a dose-dependent manner as shown by a decrease in the number of TRAP-positive multinucleated osteoclasts (Figure 2A). Notably, huge TRAP-positive multinucleated osteoclasts (nuclei ≥ 6) decreased in the zinc-treated group (Figure 2B). The TRAP activities of BMMs induced by RANKL were also inhibited by zinc in a dose-dependent manner (Figure 2C). These results suggest that zinc inhibits osteoclast formation and fusion.

### Zinc suppresses Nfatc1 expression and transcriptional/DNA binding activity

**Figure 3:**
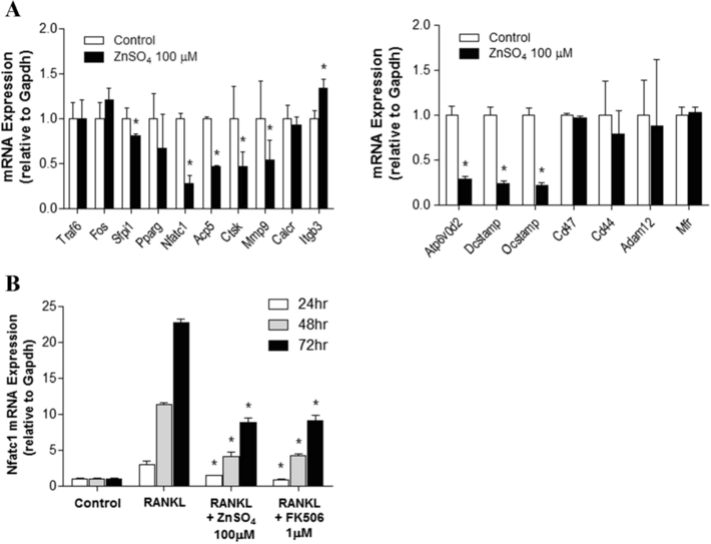
**Zinc regulates the mRNA levels of *Nfatc1 *and its target genes during BMM osteoclastogenesis. (A)** BMMs were cultured in the present of M-CSF (30 ng/ml) and RANKL (120 ng/ml) for 4 days with or without ZnSO_4_ (100 μM). In RANKL-induced osteoclasts, mRNA expression of osteoclast marker genes (left panel) and fusion-related genes (right panel) were determined using real-time PCR. The results are expressed relative to each mRNA on day 4. **(B)** BMMs were cultured with M-CSF (30 ng/ml) and RANKL (120 ng/ml) in the presence or absence of ZnSO_4_ (100 μM) and FK506 (1 μM). After 24, 48, or 72 hours, total RNA was extracted from the cultured BMMs and mRNA levels were examined using real-time PCR. Data are presented as the mean ± S.D. of three independent experiments; * p < 0.05 compared to control and RANKL, respectively.

To elucidate the molecular mechanism of zinc’s inhibition of osteoclast formation and fusion, we analyzed the mRNA levels of genes during osteoclast differentiation in the presence of zinc. Among the many genes related to osteoclast differentiation and fusion, the mRNA levels of *Nfatc1*, a master regulator of osteoclast formation, and its target genes, such as *Acp5*, *Ctsk*, *Mmp9*, *Atp6v0d2*, *Dcstamp*, and *Ocstamp*[9,25], were decreased (Figure 3A). During osteoclastogenesis periods in BMMs, the mRNA level of *Nfatc1* gradually increased due to auto-amplification. Zinc, however, suppressed *Nfatc1* mRNA expression as much as FK506, a known inhibitor of calcineurin-NFATc1 signaling during osteoclastogenesis (Figure 3B).

**Figure 4:**
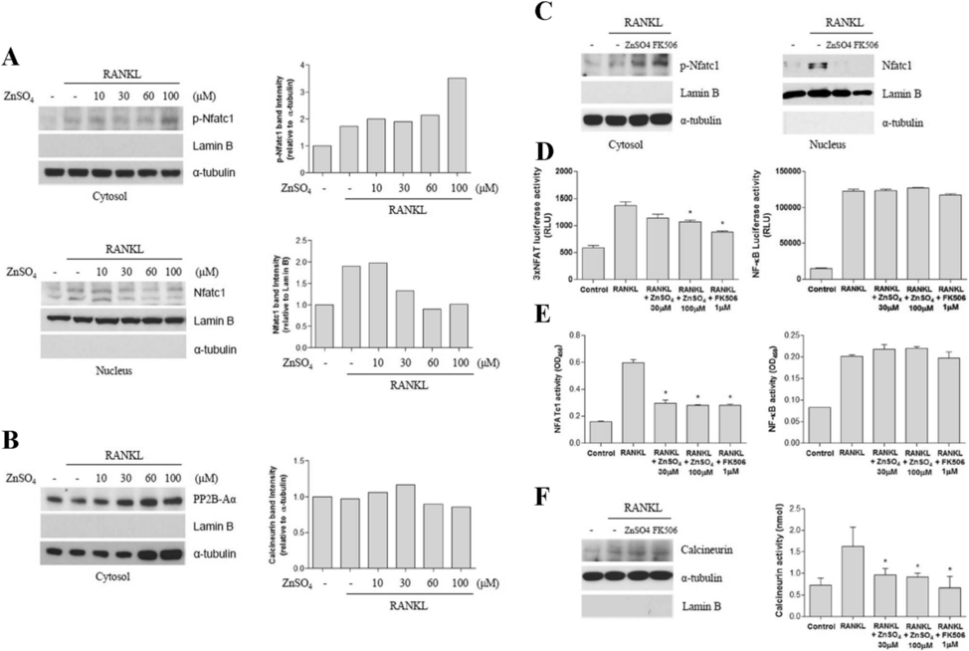
**Zinc Inhibits RANKL-induced Nfatc1 Activation by suppressing NFATc1 Translocation to the Nucleus in RAW264.7 cells. (A, B)** RAW264.7 cells were incubated with RANKL (35 ng/ml) alone or RANKL (35 ng/ml) with various concentrations of ZnSO_4_. After 30 minutes, cytosolic and nuclear fractions were extracted from each group and evaluated by western blotting with the anti-phospho-Nfatc1 antibody (**A**, upper panel and **C**, left panel), anti-Nfatc1 antibody (**A**, lower panel, **C**, right panel), or anti-PP2B-Aα antibody **(B)**, which is the catalytic subunit of calcineurin. Subcellular fraction purity and equal sample loading were evaluated by analyzing Lamin B and α-tubulin. Protein levels were quantified using densitometry. **(C)** RAW264.7 cells were incubated for 30 minutes with RANKL (35 ng/ml), RANKL (35 ng/ml) with ZnSO_4_ (100 μM), or RANKL (35 ng/ml) with FK506 (1 μM). Cytosolic phospho-Nfatc1 and nuclear Nfatc1 were analyzed using western blot. **(D, E)** RAW264.7 cells were stimulated with RANKL (R) or RANKL (R) plus ZnSO_4_ (30 or 100 μM) for 30 minutes. Nuclear fractions were prepared, and the transcriptional and DNA binding activity of Nfatc1 and NF-κB were measured using luciferase reporter assay and ELISA, respectively. RLU, Relative Light Units **(F)** RAW264.7 cells were cultured as shown in panel C. Cytosolic PP2B-Aα was examined by western blot and calcineurin activity was compared with the treated groups. Data are presented as the mean ± S.D. of three independent experiments; * p < 0.05 compared to RANKL (R).

To analyze how zinc suppresses *Nfatc1* mRNA expression, we evaluated whether zinc inhibits osteoclast differentiation signaling pathways. Calcineurin dephosphorylates cytosolic p-Nfatc1 after which the dephosphorylated Nfatc1 translocates to the nucleus. We thus evaluated the protein levels of cytosolic p-Nfatc1 and nuclear Nfatc1 in RAW264.7 cells. Zinc dose-dependently increased cytosolic p-Nfatc1. In contrast, nuclear Nfatc1 dose-dependently decreased in response to zinc (Figure 4A). As shown in Figure 4C, the expression and transcriptional activity of Nfatc1 were induced in RAW264.7 cells after exposure for 30 minutes to RANKL. Zinc significantly reduced the protein level of activated Nfatc1 as much as FK506. These results correlated with the transcriptional and DNA binding activities of Nfatc1 (Figure 4D, 4E, left panel). NF-κB transcriptional and DNA binding activities were also induced by RANKL but were not inhibited by zinc or FK506 (Figure 4D, 4E, right lower panel).

### Zinc inhibits calcineurin activity but not expression

We investigated calcineurin activity and its protein expression in the upstream Nfatc1 signaling pathway during osteoclast differentiation. After exposure to RANKL for 30 minutes in the presence or absence of zinc or FK506 in RAW264.7 cells, PP2B-Aα, the catalytic subunit of calcineurin, was unchanged in terms of protein expression (Figure 4B). However, zinc and FK506 similarly inhibited RANKL-induced calcineurin activity (Figure 4F).

### Zinc suppresses RANKL-induced Ca2+ oscillations in RAW264.7 cells without decreasing PLCγ phosphorylation

**Figure 5:**
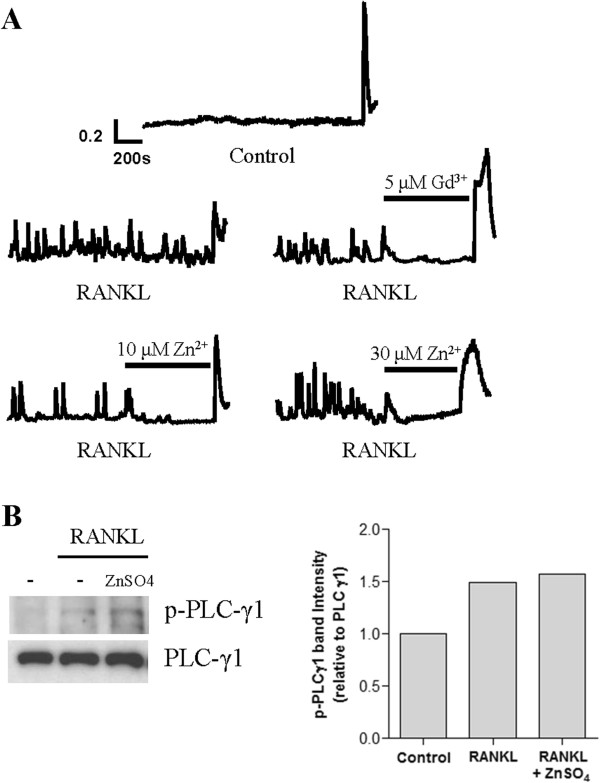
**Zinc Suppresses RANKL-induced Ca**^**2+ **^**Oscillation in RAW264.7 cells without decreasing PLCγ1 activity. (A)** RAW264.7 cells were cultured for 48 hours with RANKL (35 ng/ml) (n=3). Intracellular Ca^2+^ in single cells was measured using Fura-2/AM (5 μM). After observing RANKL-induced spontaneous Ca^2+^ oscillations for 10 minutes, ZnSO_4_ (10 or 30 μM) was added to the bath solution. At the end of the experiment, ionomycin (5 μM) was added. We used Gd^3+^, a known calcium channel blocker, as a positive control. Data shown represent one experiment of three performed with similar results. **(B)** RAW264.7 cells were stimulated for 30 minutes with RANKL (35 ng/ml) or RANKL (35 ng/ml) plus ZnSO_4_ (100 μM). Prepared proteins were analyzed by western blotting with anti-phospho-PLCγ1 or anti-PLCγ1 antibodies. Protein levels were quantified using densitometry.

Ca^2+^ oscillations in RAW264.7 cells begin at least 18 hours after RANKL stimulation during osteoclastogenesis and are sustained [9,26]. Zinc completely inhibited RANKL-induced Ca^2+^ oscillations (Figure 5A, lower panel). As a positive control, the store-operated Ca^2+^ channel blocker Gd^3+^ also curtailed RANKL-induced Ca^2+^ oscillations (Figure 5A, mid right panel). Because PLCγ activation precedes RANKL-induced Ca^2+^ oscillations, we examined the expression of the active form of PLCγ, phospho-PLCγ. Surprisingly, zinc treatment did not affect phosphorylation status of PLCγ1 in RANKL-stimulated RAW264.7 cells (Figure 5B). Based on these results, we suggest that zinc inhibits RANKL-induced Ca^2+^ oscillations independently of PLCγ1 and is involved in the Ca^2+^-calcineurin-NFATc1 signaling pathway in osteoclastogenesis.

### Nfatc1 rescues the inhibitory effects of zinc during osteoclastogenesis in RAW264.7 cell

**Figure 6:**
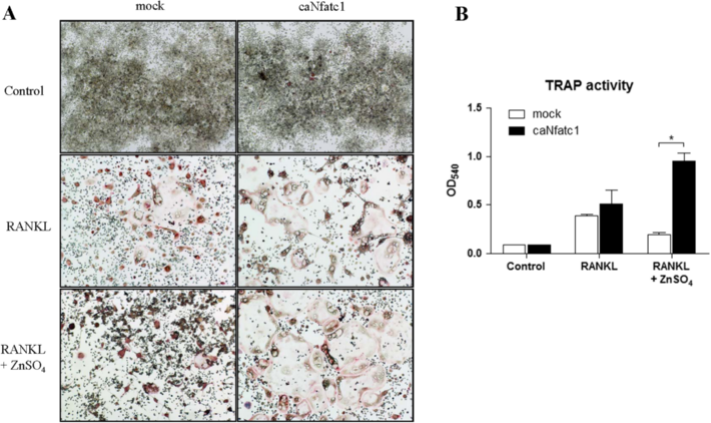
**Nfatc1 rescues the inhibitory effects of zinc during osteoclastogenesis in RAW264.7 cells. (A, B)** After electroporation with the mock (**A**, left panel) and constitutively active form of Nfatc1 (**B**, right panel), RAW264.7 cells were cultured for 4 days with RANKL (35 ng/ml) in the absence or presence of ZnSO_4_ (100 μM) (n=3). Osteoclast formation was visualized using TRAP staining in panel A. TRAP activity is shown in panel B. Data are presented as the mean ± S.D. of three independent experiments; * p < 0.05 compared to RANKL (R).

We examined whether Nfatc1 could rescue defects of osteoclastogenesis using zinc. Indeed, when we ectopically expressed a constitutively active form of Nfatc1 (caNfatc1) in RAW264.7 cells, caNfatc1 completely rescued suppression of osteoclastogenesis by zinc (Figure 6A). TRAP activity was significantly increased compared with the mock (Figure 6B). These results indicate that impairment of Nfatc1 activation is the cause of suppression during osteoclastogenesis.

## Discussion

Here we show that zinc inhibits RANKL-induced Nfatc1 translocation to the nucleus by decreasing calcineurin phosphatase activity during the early period of osteoclastogenesis. This ultimately inhibits osteoclast differentiation. Interestingly, zinc immediately diminished RANKL-induced Ca^2+^ oscillations throughout the middle or late period of osteoclastogenesis (Figure 5A) but did not suppress RANKL-induced PLCγ1 phosphorylation (Figure 5B), indicating that the inhibition of Ca^2+^ oscillations may be independent of PLCγ1. We thought that zinc could be affecting the downstream signaling pathway of the ITAM-containing adaptor-Syk-PLCγ1 axis.

After interacting with RANKL and RANK, Ca^2+^ oscillations are triggered by co-stimulatory receptors, including osteoclast-associated receptor (OSCAR), paired immunoglobulin-like receptor (PIR)-A, triggering receptor expressed on myeloid cell 2 (TREM2), and signal-regulatory protein (SIRP) β1 [22]. OSCAR and PIR-A recruit FcRγ adaptor proteins, whereas TREM2 and SIRPβ1 pair with DAP12 adaptor proteins, resulting in spleen tyrosine kinase (Syk) activation, followed by PLCγ1 phosphorylation and subsequently, Ca^2+^ influx and oscillations [27]. To maintain the Ca^2+^ oscillations, store-operated Ca^2+^ entry (SOCE) is necessary to refill the intracellular Ca^2+^ stores [28]. It was reported that 2-aminoethoxydiphenyl borate (2-APB) and SKF-96365, SOC channel blockers, significantly decrease osteoclastic survival and bone resorption [29]. Additionally, Gd^3+^, a SOC channel blocker, rapidly inhibits Ca^2+^ oscillations [30]. However, there is controversy regarding whether zinc blocks the SOC channel. Tibbits et al. reported that zinc blocked SOC channels in human salivary cell lines, human neutrophils, and rabbit cardiomyocytes [31-34]. Ambudkar et al. subsequently reported that SOC channels were not inhibited by zinc in human salivary cell lines [35-37]. In osteoclasts, zinc may act as a SOC channel blocker similar to Gd^3+^. To verify that zinc is a SOC channel blocker in osteoclasts, further studies will be needed.

Zinc is known to stimulate osteoclast apoptosis, which is mediated through Ca^2+^ signaling [38]. We first defined a concentration of ZnSO_4_ (100 μM) that was nontoxic to both BMMs and RAW264.7 cells. Although 100 μM ZnSO_4_ significantly inhibits TRAP activity in osteoclasts (Figure 2C), this concentration of zinc does not affect BMM viability (Figure 1A). As shown in Figure 2B, the number of fused multinucleated osteoclasts significantly decreased upon zinc treatment, indicating that zinc affects the fusion of multinucleated cells in BMMs. These findings suggest that the inhibitory effect of zinc on osteoclast differentiation was not caused by zinc cytotoxicity. In Figure 3B, mRNA levels of Nfatc1 decreased due to zinc and FK506 treatments as osteoclast differentiation progressed. Yet there were still some Nfatc1 inductions at 48 and 72 hours after the zinc and FK506 treatments. Asagiri et al. reported that an autoamplification of Nfatc1 was essential in osteoclast differentiation [39]. As shown in Figure 4D, the inhibitory effects of zinc and FK506 for Nfatc1 transcriptional activity were not 100%. There was some residual activity. We thought that while some autoamplification of Nfatc1 caused part of the Nfatc1 inductions after zinc and FK506 treatments, it might be not enough for osteoclast maturation.

There were many reports that zinc can inhibit calmodulin, which is an important activator of calcineurin. Brewer reported that zinc inhibits calmodulin in the erythrocyte [40]. Zinc inhibits calmodulin by competing with Ca^2+^ binding to calmodulin, which has also resulted in a conformational change of the protein [40-43]. The phosphorylation and activity of calmodulin-dependent protein kinase II is modulated by zinc as well [44]. One in vivo study showed that calmodulin level decreased in epidermal cells after intraperitoneal or intradermal zinc injections [45]. Another study showed that zinc treatment reduced calmodulin in adipocytes of obese mice [46]. We found that zinc decreased the activity of calcineurin in the early period of osteoclastogenesis of RAW264.7 cells (Figure 4F). Our results were consistent with a previous article that showed Nfatc1 translocation to the nucleus and an activation of calcineurin within 30 to 40 minutes after RANKL stimulation in RAW264.7 cells [47]. In addition, calcium which comes from intracellular calcium storage, such as the sarcoplasmic reticulum, may be working as a second messenger in the early period of osteoclast differentiation. Thus, we thought that zinc might inhibit calcineurin activity by suppression of calmodulin as shown in previous reports [40-46]. Subsequently, Nfatc1 would not be able to change into its active form and stays in the cytosol. When we overexpressed constitutively active NFATc1 in RAW 264.7 cells, the inhibitory phenotype for osteoclasts rescued (Figure 6). Since the constitutively active NFATc1 lacks phosphorylation sites in the regulatory domain, it would be expected to effects of zinc on NFATc1 kinases such as calcineurin which is the most important NFATc1 kinase. So, we thought that the rescue of the phenotype was caused by calcineurin-NFATc1 pathway. But, we cannot exclude effects of zinc on other modulators of NFATc1 pathway.

It was previously reported that zinc treatment for 24 hours suppresses RANKL-induced NK-κB luciferase activity in RAW264.7 cells [48]. It was also reported that zinc supplementation for 3 months decreases the DNA binding capacity of NF-κB in mononuclear cells from sickle cell disease patients [49]. These results differed from our own results that zinc did not inhibit NK-κB transcriptional activity in RAW264.7 cells (Figure 4D). This difference may be controversial. In general, however, an efficiency of DNA transfection in RAW264.7 cells is very poor. Thus, we used electroporation for more efficient gene expression instead of chemical transfections and increased the efficiency up to 65%. We thought that this may be a cause of the difference. Also, Hie et al. demonstrated that Zn treatment could inhibit RANK expression during osteoclast differentiation [50], but its molecular mechanism was unclear. Further investigation is needed in future studies.

Intracellular zinc signaling consists of two signaling pathways. The early zinc signal, which is transcription-independent, is rapidly induced by an extracellular stimulus, such as FcϵRI [16]. Late zinc signaling involves transcription-dependent changes in expression of zinc transporters, such as ZIP (SLC30A) and ZnT (SLC39A) [16,51-53]. Zinc transporters are ubiquitously expressed and play a role in maintaining the levels of cellular zinc by controlling its influx, efflux, and sequestration. Zinc signaling also modulates numerous cellular processes involved in cell differentiation, proliferation, and growth [54]. Because zinc transporters are expressed in osteoclasts and some are up-regulated during osteoclast differentiation, zinc may play an important role in osteoclast differentiation [55,56].

FK506, an immunosuppressant, is a potent inhibitor of calcineurin phosphatase activity. It inhibits both bone resorption and formation [57]. Overall, FK506 is not beneficial for increasing bone mass and quality. Zinc, on the other hand, inhibits osteoclastogenesis as well as stimulates bone formation in mice and rats [38,48]. In particular, we also found that zinc stimulates osteoblastogenesis in human mesenchymal stem cells (data not shown). Thus, if zinc could be effectively transferred in bone tissue, it may be beneficial for increasing bone mass and quality.

## Conclusions

**Figure 7:**
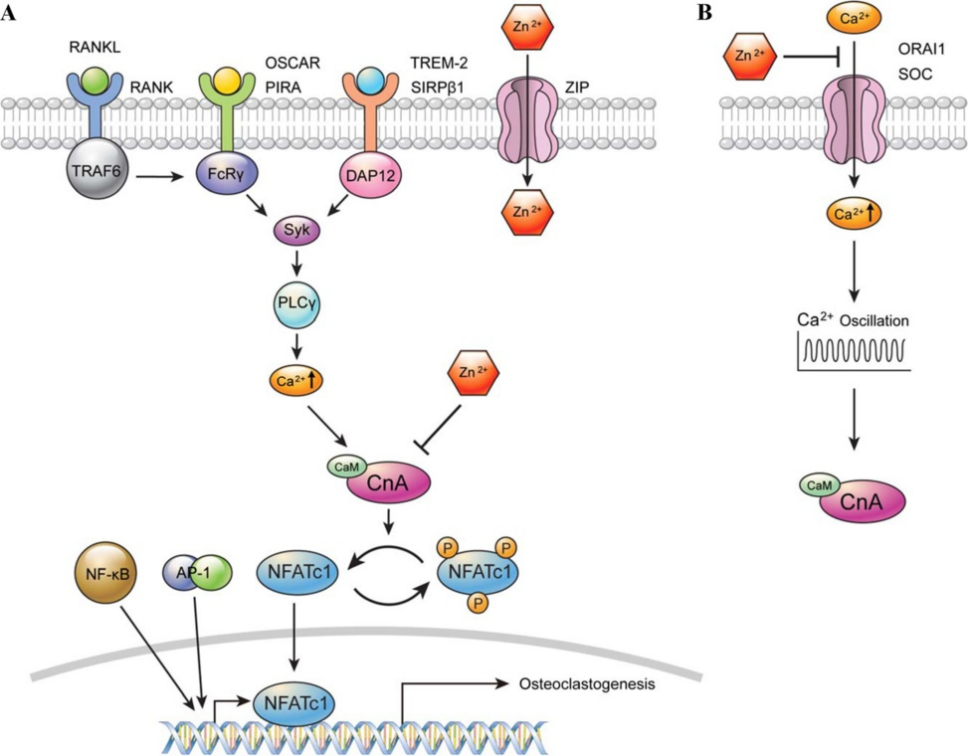
**The proposed molecular mechanism for the inhibitory effects of zinc on RANKL-induced osteoclastogenesis.** Schematic models of the inhibitory effects of zinc on the Ca^2+^-Calcineurin-NFATc1 signaling pathway; **(A)** Zinc may inhibit calcineurin in the cytosol in the early period of osteoclastogenesis. **(B)** In the middle or late period, zinc could be suppressing calcium oscillations by blocking calcium influx from extracellular space. Cn A, Calcineurin A subunit; CaM, Calmodulin; P, phosphorylated.

We have shown that zinc is an important inhibitory modulator during osteoclast differentiation that acts on the Ca^2+^-Calcineurin-NFATc1 signaling pathway. We proposed molecular mechanisms through which zinc may inhibit calcineurin in at least the early period of osteoclast differentiation and inhibit calcium oscillations in the middle or late period of osteoclast differentiation (Figure 7). Therefore, zinc might be a good therapeutic candidate for preventing osteoporosis and arthritis caused by NFATc1 activation in osteoclasts.

## Materials and methods

### Cell culture and reagents

Primary cultured mouse BMMs (bone marrow-derived monocytes) and RAW264.7 cells (Korean Cell Line Bank, South Korea) were respectively cultured in α-minimum essential media (α-MEM, Gibco) and Dulbecco’s modified Eagle’s media (DMEM, Thermo) supplemented with 10% fetal bovine serum (FBS, Gibco) in 5% CO_2_ at 37°C. M-CSF and RANKL were purchased from KOMA Biotech (South Korea) and ATGen (South Korea), respectively. The monoclonal antibody for α-tubulin and polyclonal antibodies for p-Nfatc1 (Ser259), Nfatc1, PP2B-Aα and Lamin B were obtained from Santa Cruz Biotechnology (Santa Cruz, CA). Polyclonal antibodies for p-PLCγ1 (Tyr783) and PLCγ1 were procured from Cell Signaling Technology (Beverly, MA). Fura-2/AM was purchased from Teflabs (Austin, TX). Zinc sulfate (Zn^2+^), gadolinium chloride (Gd^3+^) and FK506 were obtained from Sigma-Aldrich (St Louis, MO). Constitutively active Nfatc1 plasmid vector was generously gifted by Dr. Anjana Rao [58].

### Preparation of BMMs and in vitro osteoclastogenesis

The femur and tibia were removed from 6-week-old male C57BL/6 mice. Cells derived from the bone marrow were collected and cultured in growth media containing M-CSF (10 ng/ml). After 24 hours, nonadherent cells were collected and seeded in a 100 mm dish and treated with M-CSF (30 ng/ml). After 48 hours, nonadherent cells were washed and the adherent cells were used as BMMs. BMMs were detached from the 100 mm dish using Detachin^TM^ (Genlantis, San Diego, CA). The obtained cell pellet was resuspended and seeded on dishes or plates for osteoclastogenesis. BMMs (1 × 10^5^ cells/ml) were cultured for 4 days in growth media containing M-CSF (30 ng/ml) and RANKL (120 ng/ml) with or without ZnSO_4_. Also, RAW264.7 cells were cultured for 4 days in growth media containing RANKL (35 ng/ml) with or without ZnSO_4_ for osteoclastogenesis. For rescue experiments, RAW264.7 cells were transfected with constitutively active Nfatc1 plasmid by electroporation using the Amaxa Cell line Nucleofector™ kit V (Lonza).

### Cell viability assay

RAW264.7 cells were maintained in growth media with or without ZnSO_4_ (10, 30, 60, 100, 200, or 500 μM) for 24 hours. Additionally, BMMs were cultured in growth media containing M-CSF (30 ng/ml) in the presence or absence of ZnSO_4_ (10, 30, 60, 100, 200, or 500 μM) for 4 days. Cell viability assays were performed using an EZcytox cell viability assay kit (Daeillab Service, South Korea) according to the manufacturer’s instructions. Briefly, the cells were plated in 96-well plates at 1 × 10^4^ cells per well and cultured in growth media. At the indicated time points, cells were incubated for 4 h at 37°C with WST-1. The number of viable cells in triplicate wells was measured at an absorbance wavelength of 450 nm.

### Measurement of TRAP activity and TRAP staining

TRAP activity was measured from osteoclast culture supernatants using a TRAP Staining kit (Kamiya Biomedical Company). Supernatants (30 μl) were incubated for 3 hours at 37°C with 170 μl of the chromogenic substrates in a tartrate-containing buffer. TRAP activities were measured in terms of the absorbance at a wavelength of 540 nm. TRAP was stained using a similar method as described above. Cultured cells were incubated with a fixative for 5 minutes at room temperature, washed with distilled water, and treated for 20 minutes at 37°C with the chromogenic substrate in tartrate-containing buffer. After staining, TRAP-positive multinucleated (nuclei ≥ 3) cells were counted using manual counting or a nuclei counter plug-in in the Image J program.

### Real-time reverse transcription-PCR

RNA was extracted from BMMs on the indicated days using TRIZOL reagent (Invitrogen, Carlsbad, CA). cDNA was reverse transcribed using random hexamers and SuperScript-III reverse transcriptase (Invitrogen). The cDNA was used in real-time PCR with a KAPA SYBR FAST ABI Prism qPCR kit (Kapa Biosystems). The specific primer pairs are shown in Table 1. Nfatc1 and other mRNAs were measured using a StepOne (Applied Biosystems) Real-Time PCR System. The PCR program was initiated for 20 seconds at 95°C, followed by 40 thermal cycles of 3 seconds at 95°C and 30 seconds at 60°C, and terminated for 15 seconds at 95°C, 1 minute at 60°C, and 15 seconds at 95°C. Data were analyzed according to the comparative cycle threshold (Ct) method [59] and were normalized to GAPDH in each sample. We examined individual gene expression in triplicate and repeated each experiment more than three times.

**Table 1:** List of primer sequences

**Transcript**	**Primer sequence (5′ → 3′)**
*Nfatc1*	F: GGTAACTCTGTCTTTCTAACCTTAAGCTC
	R: GTGATGACCCCAGCATGCACCAGTCACAG
*Traf6*	F: GAAGAGGTCATGGACGCCAA
	R: CGGGTAGAGACTTCACAGCG
*Fos*	F: GGAGAATCCGAAGGGAACGG
	R: GCAATCTCAGTCTGCAACGC
*Sfpi1*	F: CAGCAGCTCTATCGCCACAT
	R: ATCCGGGGCATGTAGGAAAC
*Pparg*	F: ATTGAGTGCCGAGTCTGTGG
	R: GGCATTGTGAGACATCCCCA
*Acp5*	F: GGGAAATGGCCAATGCCAAAGAGA
	R: TCGCACAGAGGGATCCATGAAGTT
*Ctsk*	F: AGGCAGCTAAATGCAGAGGGTACA
	R: ATGCCGCAGGCGTTGTTCTTATTC
*Mmp9*	F: CGCTCATGTACCCGCTGTAT
	R: TGTCTGCCGGACTCAAAGAC
*Calcr*	F: TGCGGCGGGATCCTATAAGT
	R: TGGTTGGCACTATCGGGAAC
*Itgb3*	F: TTACCACGGATGCCAAGACC
	R: CCCCAGAGATGGGTAGTCCA
*Atp6v0d2*	F: GGCTGTGCTGGTTGAAACAC
	R: TAACAACCGCAACCCCTCTG
*Dcstamp*	F: TCCTCCATGAACAAACAGTTCCAA
	R: AGACGTGGTTTAGGAATGCAGCTC
*Ocstamp*	F: ATGAGGACCATCAGGGCAGCCACG
	R: GGAGAAGCTGGGTCAGTAGTTCGT
*Cd47*	F: GTGGTTGTTGGAGCCATCCT
	R: TGCCATGATGCAGAGACACA
*Cd44*	F: CAACCGTGATGGTACTCGCT
	R: TTGAGTGCACAGTTGAGGCA
*Adam12*	F: CATCCAGACGTGCTGACTGT
	R: AGCTGGGACGAGTTTGTAGC
*Mfr*	F: TGGCTTCTCTCCCCGGAATA
	R: CCTCGGGGTAGAACCTCTCA

### Cell fractionation

RAW264.7 cells at 70–80% confluence were incubated in α-MEM containing RANKL (35 ng/ml), with or without ZnSO_4_, for the indicated times, washed, and scraped in cold PBS. Cell pellets were fractionated into cytoplasmic and nuclear fractions using a NE-PER Nuclear and Cytoplasmic Extraction Reagents kit (Pierce, Rockford, IL).

### Western blots

Cell lysates were prepared using radioimmunoprecipitation assay (RIPA) buffer [50 mM Tris-Cl (pH 7.4), 150 mM NaCl, 1% NP-40, 0.25% Na-deoxycholate, 0.1% SDS with 1 mM EDTA (pH 8.0), 1 mM phenylmethylsulfonyl fluoride, 2 μg/ml aprotinin, 2 μg/ml leupeptin, 4 mM Na3VO4, and 10 mM NaF]. The samples (10–30 μg protein/well) were resolved using SDS–PAGE (6-10% gels), and proteins were transferred to nitrocellulose membranes. The membrane was blocked in 5% skim milk and incubated with antibodies against p-Nfatc1 (1:3000), Nfatc1 (1:4000), PP2B-Aα (1:500), p-PLCγ1 (1:1000), PLCγ1 (1:1000), α-tubulin (1:500), and lamin B (1:1000). This procedure was followed by incubation with a horseradish peroxidase-conjugated secondary antibody for 1 hour. Chemiluminescence was detected using an ECL system (GE Healthcare).

### Nfatc1 and NF-κB transcriptional activity

Nfatc1 and NF-κB transcriptional activities were measured using luciferase reporter assay. Luciferase reporter gene plasmids were transfected in RAW264.7 cells using the Amaxa Cell line Nucleofector™ kit V (Lonza). pRL-TK (Promega) was used as a normalization control to check transfection efficiency. The next day, cells were stimulated in RANKL with or without zinc sulfate or FK506. The cells were collected 24 hours after treatment and lysed with 1 × Passive lysis buffer (Promega). Luciferase activity was measured using the Dual-Luciferase Reporter Assay System (Promega).

### Nfatc1 and NF-κB DNA binding activity

Nfatc1 and NF-κB (p65) DNA binding activities were measured using a TransAM transcription factor enzyme-linked immunosorbent assay (ELISA) kit (Active Motif, Carlsbad, CA). Nuclear extracts (5 μg) were incubated for 30 minutes at room temperature on Nfatc1 and NF-κB consensus oligonucleotide-coated ELISA plates. Activated transcription factors bound to consensus oligonucleotides were detected using a specific antibody and measured at 450 nm.

### Calcineurin activity

Cellular calcineurin phosphatase activity was measured in cell extracts using a Calcineurin Cellular Activity assay kit (Enzo Life Sciences, Farmingdale, NY). In brief, cells were lysed in a lysis buffer containing protease inhibitors and centrifuged. The same amount of protein (5 μg) was used in the calcineurin activity assays. Colorimetric measurements were performed at 620 nm. The amount of phosphate released by calcineurin was calculated using a standard curve.

### Intracellular Ca^2+^ measurement

RAW264.7 cells were seeded on a cover glass in a 35 mm dish (1 × 10^5^ cells per dish) and activated for 48 hours with RANKL (35 ng/ml). Cells were incubated for 30 minutes at room temperature with 5 μM Fura-2/AM and 0.05% Pluronic F-127 (Invitrogen) and washed with a bath solution (140 mM NaCl, 5 mM KCl, 1 mM MgCl_2_, 10 mM HEPES, 1 mM CaCl_2_, 10 mM glucose, 310 mOsm, pH 7.4). The cover glass was transferred to a perfusion chamber, and the cells were continuously perfused with prewarmed (37°C) bath solution. The excitation wavelengths for Fura-2 fluorescence were 340 and 380 nm and the emission wavelength was 510 nm. The fluorescence intensity was measured by the ratio of emitted fluorescence (F_340_/F_380_), which was monitored using a CCD camera (Universal Imaging Co., Downingtown, PA) every 2 seconds. CCD camera images were analyzed using MetaFluor software (Universal Imaging, Downingtown, PA). For the inhibition assays, ZnSO_4_ or a known store-operated Ca^2+^ (SOC) channel inhibitor, Gd^3+^ was added 10 minutes after RANKL-induced Ca^2+^ oscillations. At the end of the assay, 5 μM ionomycin (Sigma) was added.

### Statistical analysis

The results are shown as the mean ± standard deviation (S.D.) from at least three independent experiments. The differences between groups were analyzed using Student’s t-tests and p < 0.05 was considered statistically significant.

## Abbreviations

2-APB: 2-aminoethoxydiphenyl borate; BMMs: Bone marrow-derived monocyte cells; GEO: Gene expression omnibus; Nfatc1: Nuclear factor of activated T-cells, cytoplasmic 1; OSCAR: Osteoclast-associated receptor; PIR: Paired immunoglobulin-like receptor; p-Nfatc1: Phospho-Nfatc1; p-PLCγ1: Phospho-phospholipaseCγ1; RANKL: Receptor activator of NF-κB ligand; RLU: Relative light units; SIRP: Signal-regulatory protein; SOC: Store-operated Ca^2+^; SOCE: Store-operated Ca^2+^ entry; Syk: Spleen tyrosine kinase; TRAP: Tartrate-resistant acid phosphatase; TREM2: Triggering receptor expressed on myeloid cell 2.

## Competing interests

The authors declare that they have no competing interests.

## Authors’ contributions

KHP wrote the manuscript, performed most of the experiments, analyzed data, and obtained IACUC approval. BP and DMS performed calcium oscillation assays and analyzed the data. DSY, SK and JWL reviewed and helped in the data analyses. HGL, JS and JHP critiqued with the drafting and preparing of the manuscript for publication. JML conceived and designed the study and wrote the manuscript. All authors read and approved the final manuscript.

## Supplementary Material

Additional file 1: Table S1 List of Zinc-related genes that were up-regulated (log_2_ ratio > 4.0) during osteoclastogenesis. Click here for file
