# Supplementary material for: Zinc inhibits osteoclast differentiation by suppression of Ca2+-Calcineurin-NFATc1 signaling pathway
Source: Cell Commun Signal. 2013 Oct 2;11:74. doi: 10.1186/1478-811X-11-74 (PMC3851046; doi:10.1186/1478-811X-11-74)
Supplement: Additional file 1: Table S1 — List of Zinc-related genes that were up-regulated (log2 ratio > 4.0) during osteoclastogenesis. [file 1478-811X-11-74-S1.doc]

**Additional file 1: Table S1. List of Zinc-related genes that were up-regulated (log_2_ ratio > 4.0) during osteoclastogenesis**

| GeneSymbols | GeneNames | Log_2_ ratio * | Log_2_ ratio ** | Gene Ontology (GO) | | |
| --- | --- | --- | --- | --- | --- | --- |
|  |  |  |  | Biological  process | Cellular  component | Molecular  function |
| *Mmp9* | Matrix metalloproteinase-9 | 5.69 | 8.93 | - | - | Zinc ion binding |
| *Car2* | Carbonic anhydrase 2 | 4.16 | 6.93 | Response to  zinc ion | - | Zinc ion binding |
| *Mt3* | Metallothionein-3 | 4.13 | 6.82 | Cellular zinc ion homeostasis,  Zinc ion transport | - | Zinc ion binding |

*: Zhao et al. , **: Miyauchi et al.
